# Ontological Knowledge Engine and Health Screening Data Enabled Ubiquitous Personalized Physical Fitness (UFIT)

**DOI:** 10.3390/s140304560

**Published:** 2014-03-07

**Authors:** Chuan-Jun Su, Chang-Yu Chiang, Meng-Chun Chih

**Affiliations:** 1 Department of Industrial Engineering & Management, Yuan Ze University, No.135, Yuandong Rd., Zhongli City, Taoyuan County 320, Taiwan; E-Mail: s978905@mail.yzu.edu.tw; 2 Project Management, Shun On Electronic Co. Ltd, 6F., No.22, Taiyuan St., Zhubei City, Hsinchu County 302, Taiwan; E-Mail: Baby1121tw@gmail.com

**Keywords:** physical fitness, personalized exercise plan, Health Level Seven International (HL7), knowledge-based system, REST style web services, ontology

## Abstract

Good physical fitness generally makes the body less prone to common diseases. A personalized exercise plan that promotes a balanced approach to fitness helps promotes fitness, while inappropriate forms of exercise can have adverse consequences for health. This paper aims to develop an ontology-driven knowledge-based system for generating custom-designed exercise plans based on a user's profile and health status, incorporating international standard Health Level Seven International (HL7) data on physical fitness and health screening. The generated plan exposing Representational State Transfer (REST) style web services which can be accessed from any Internet-enabled device and deployed in cloud computing environments. To ensure the practicality of the generated exercise plans, encapsulated knowledge used as a basis for inference in the system is acquired from domain experts. The proposed Ubiquitous Exercise Plan Generation for Personalized Physical Fitness (UFIT) will not only improve health-related fitness through generating personalized exercise plans, but also aid users in avoiding inappropriate work outs.

## 1. Introduction

Technological advancement in economics and medical knowledge has increasingly raised the public awareness of health issues for promoting longevity and quality of life. While standards of living have improved with the rise of technology, stresses and deteriorated environmental factors such as air and noise pollution lead to an increase of “sub-health” phenomena [1]. As defined by the World Health Organization (WHO), sub-health is a state between health and disease when all necessary physical and chemical indexes are tested negative by medical equipment, and things seem normal, but the person experiences all kind of discomfiture and even pain. For example, a “healthy” person who lacks flexibility or muscular endurance could easily be injured by lifting a heavy box, and thus would actually be considered to be sub-healthy. A global research conducted by the WHO shows that about 75 percent of the World's population are “sub-healthy”, while only 20 and 5 percent are classified as morbid and healthy, respectively [2]. Sub-health and its related factors require urgent attention in the area of modern preventive medicine.

Regular exercise generally leads to better health-related fitness and thus improves sub-health conditions [3]. Fitness is composed of five components: (1) body composition; (2) cardiovascular endurance; (3) flexibility; (4) muscular endurance; and (5) muscular strength [4]. These components are important indicators of the relative benefits of various physical activities and of a person's state of health. Improved fitness not only makes it easier to maintain physical health but also makes the body less susceptible to common diseases.

Although a basic understanding of the principles of exercise can be obtained from books or the Internet, ideal fitness programs vary by individual according to personal health conditions and medical history. Applying a “one size fits all” approach to fitness may be harmful to a person's health. However, it is generally time-consuming and costly for consulting domain professionals to compile a personalized exercise plan. Moreover, the professionals may recommend inconsistent exercise plans which may lead to some unpredictable side effect by pulling their information from a variety of sources and favored practices. A knowledge-based system which encompasses domain-expert knowledge and is capable of furnishing consistent exercise plans based on users' current health and medical conditions may successfully address these issues.

Ontology is an emerging technology which enables the advanced representation, management, and sharing of knowledge. It is also central to many applications in fields including information management, systems integration and semantic web services *etc.* [5]. There are in general three major uses of ontology: (1) to assist in communication between human beings; (2) to achieve interoperability (communication) among software systems [6]; and (3) to improve the design and the quality of software systems [7]. With the features and results of related studies, such as [8,9], ontology technology presents great potential to be applied in building exercises representation and the reasoning of personalized exercise plan based on users' physical fitness test results and health screening data. Through an ontology-based knowledge engine, cross-domain knowledge solicited from human experts can be visualized and applied to reasoning and modeling [10].

To ensure the interoperability of electronic health data (for fitness and health screening) in an ontological knowledge engine, a uniform medical information standard Health Level Seven (HL7) was adopted. As a global authority on standards for the sharing, integration, and retrieval of electronic health information, HL7 responds to the increased demand in healthcare interoperability that enhances care delivery, optimizes workflow and augments knowledge transfer [11,12].

The main objective of this research is to develop an ontology driven knowledge-based system for generating specifically designed exercise plan based on: (1) the user profile; (2) the HL7-based data of user's physical fitness; and (3) the HL7-based data of user's health screening to improve physical fitness and to make the body less prone to common diseases. The generated exercise plan can be accessed ubiquitously by using any Internet-enable device through the paradigm of REST service. In order to generate a personalized exercise plan which is pragmatic, the encapsulated knowledge used for inference in the system is acquired from the domain professionals.

In this paper, we present the development of an ontology-driven knowledge-based system UFIT for generating custom-designed exercise plans based on a user's profile and health status, incorporating international standard Health Level Seven International (HL7) data on physical fitness and health screening. The generated plan can be published as Representational State Transfer (REST)-style web services which can be accessed from any Internet-enabled device and deployed in cloud computing environments. The developed UFIT serves as an intelligent system which supports not only improving physical fitness of individuals through furnishing appropriate exercise plans but also avoiding inappropriate workouts. The remainder of the paper is organized as follows: in Section 2 we discuss studies related to ontology, HL7, and ubiquitous computing. The design and framework of the knowledge-based system which includes the ontology-based knowledge engine is presented in Section 3. In Section 4 we illustrate major implementation details including the system configuration and implementation of a sample scenario. Finally, in Section 5 we provide conclusions and suggestions for future research.

## 2. Related Works

### 2.1. Ontology-Driven Personalized Recommendations

An ontology is defined as a formal, explicit specification of a shared conceptualization. It describes the concepts and relationships that are important in a particular domain, providing a vocabulary for that domain as well as a computerized specification of the meaning of terms used in the vocabulary [13,14]. Ontology technology has been broadly applied to perform knowledge modeling and generate personalized information or recommendation in various fields, such as e-commerce [15], e-Library [16], e-learning [17], biomedicine [18,19], *etc*.

Kanellopoulos developed an intelligent web portal that can help people living in Europe find airline seats that match their personal travelling preferences. An ontology-guided search engine was defined for providing intelligent matches between airline seats offers and travelling preferences [15]. Liao *et al.* designed and implemented an ontology-based personalized recommendation system for library services. A personal ontology was developed for representing a unique user interest on specific subjects to filter out unsuitable recommendations [16]. Vesin *et al.* presented how ontology can be used in building personalized learning paths, maintaining up to date learners' cognitive states, and providing adaptation process in e-learning systems [17]. Puustjärvi, J. and Puustjärvi, L. developed a Personal Health Server that allows the interoperation of e-health tools through a shared ontology. The shared ontology was built by integrating the ontologies of the e-health tools for supporting the interoperability of personal health records, e-health oriented blogs, and information therapy [18,19].

The aim of ontology is to formalize domain knowledge in a generic way and provide a common understanding of a domain, which may be used and shared by applications and groups. It helps realize reasoning and can be used in generating personalized recommendations [20].

### 2.2. Ontology-Based Healthcare Systems

In the domain of healthcare, ontologies have been recognized as a key technology in helping to furnish the semantics required for deriving proper treatment through integrating clinical guidelines [21]. Valls *et al.* conducted a work towards a common understanding of the healthcare organizations by using the ontological paradigm to model the organizational knowledge [22]. The work also addressed the issue of exploiting ontology in a special healthcare application of provisioning home care for the patients with chronic diseases. The K4CARE project was presented by Riaño *et al.*, which utilized an ontology to model knowledge required in supporting the clinical decision making of health care providers [19]. The ontology was used to model the health-care concepts related to the required home care of chronically ill patients as well as the personalization of health-care knowledge to aid clinical decisions.

Medical differential diagnosis is based on the estimation of multiple distinct parameters to determine the most probable diagnosis. García-Crespo *et al.* proposed an ontology-driven medical diagnosis system, ODDIN, which uses ontologies representing specific structured information, computation of probabilities of various factors, and logical inferences of the patient's signs and symptoms. ODDIN may serve as a training tool for decision-making by medical personnel and students [23].

**Figure 1.**
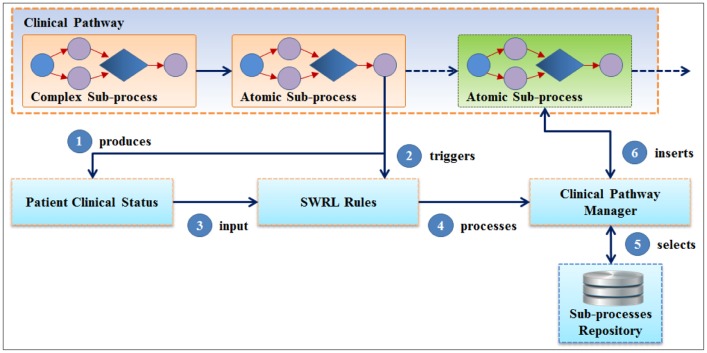
SEMPATH adaptation methodology [24].

Alexandrou *et al.* described an ontological software platform called SEMPATH, which can offer personalized treatment plans by using and managing health care business processes (clinical pathways). During the execution of clinical pathways, the system considers the patient's clinical status and reaction to the treatment scheme according to the SWRL rules in reconfiguring the next treatment steps [24]. A meta-model clinical pathway based adaptation methodology used in SEMPATH is illustrated in Figure 1. The adaptation methodology was adopted for developing our Ontological Knowledge Engine (UOKE) to determine the sequence of process execution steps that occur during the service provision.

### 2.3. Ontology and HL7 Standard

Interoperability is one of the most essential requirements for health care systems to reach the benefits promised by adopting HL7-based systems and Electronic Medical Records (EMRs). There are significant numbers of methodologies and architectures developed to address the issues of interoperability of the coalition's systems in recent years [25]. Slavov *et al.* proposed an HL7-compliant data exchange software tool called Collaborative Data Network (CDN) aiming for clinical information sharing and querying [26]. The clinical documents in CDN are modeled in compliant with HL7 v3 standard and encoded in eXtensive Markup Language (XML) format, which can be ultimately deployed in a cloud environment to support large-scale management and vast amounts of clinical data sharing [26].

The integration of HL7 standards and ontology technology has been widely applied in supporting system interoperability among applications in the medical domain [27]. By assimilating HL7-compliant clinical message with ontology, Orgun and Vu developed an electronic Medical Agent System (eMAGS) to facilitate the flow of patient information across health care organizations [28]. A simulation framework and computational test-bed was proposed by Argüello Casteleiro *et al.* for supporting simulations of clinical situations that boosted the integration between HL7 and ontology to achieve content layer data modeling and interoperability between online clinical cases and medical guidelines [29]. Similar works are further elaborated in [30].

**Figure 2.**
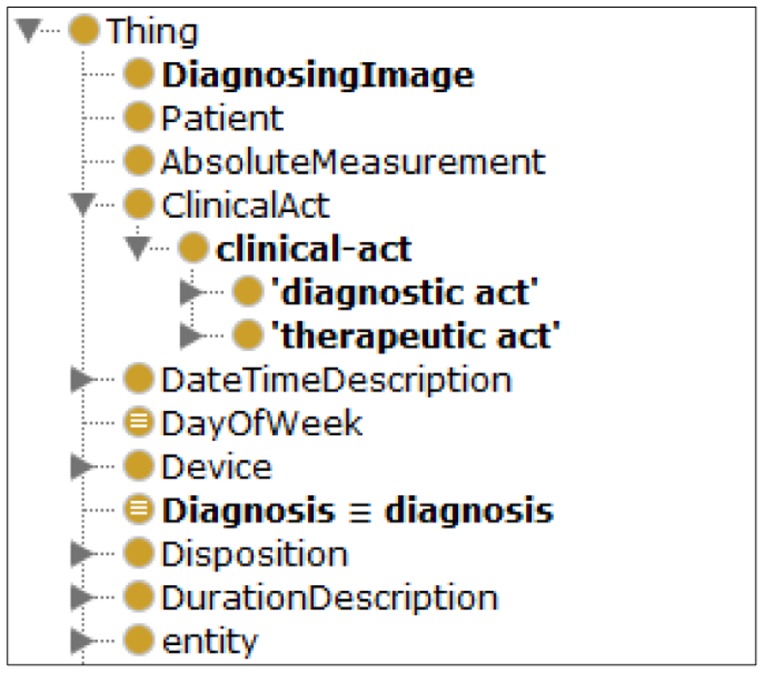
The partial classes of the “Problem-Oriented Medical Record Ontology” in HL7-sample-plus-owl.

As a core component of the proposed UFIT, the knowledge engine was built on top of problem-oriented medical record ontology “HL7-sample-plus-owl” defined by World Wide Web Consortium (W3C) as illustrated in Figure 2 [31]. According to W3C, “The goal of HL7-sample-plus-owl is to define a minimal set of terms that connect representations from well-defined healthcare information and process models with more expressive foundational ontologies through the use of the criteria outlined in the traditional problem-oriented medical record structure.” To ensure ubiquitous accessibility and wide area interoperability, we designed and developed UFIT, an HL7-compliant system driven by an ontology-based knowledge engine founding on HL7-sample-plus-owl. UFIT is capable of processing user health screening data and personal information from any HL7-enabled medical organization and subsequently generates personalized exercise plans.

### 2.4. REST Style Web Services for Building Ubiquitous Web Services

Representational State Transfer (REST) is a pattern of resource operations that is introduced by Roy Fielding as architectural style for distributed hypermedia systems, describing the software engineering principles and the interaction constraints [32]. Different from the complex SOAP (Simple Object Access Protocol)-based architecture and standard, REST is a simpler approach to achieve loosely-coupled and interoperable delivery of web services. REST is simpler method that along with its natural fit over HTTP and contributed to its status as a method for Web 2.0 applications to expose their data [33].

A web service is a technology and platform-independent architecture where loosely coupled components communicate through interfaces over standard web protocols. It may be composed at run-time to accomplish complex tasks featured with dynamic, flexible, and heterogeneous communication [34]. It allows heterogeneous devices to interact among one another over the web. A ubiquitous web service is referred to as a web service which can be accessed pervasively through well-defined Internet protocol. It adapts the Web to user preferences and the dynamic circumstances of devices and environment.

The REST services approach provides an efficient way to cope with the highly complex computing demand and intensive information provisioning in ubiquitous environments [35,36]. For example, a multi-domain context-aware service platform CUBIQ (Cross UBIQuitous platform) was established in Japan using REST services. The platform enables heterogeneous devices, sensors, mobile phones, and actuators, to be integrated and universally functioned [37]. Guinard *et al.* developed an interesting REST-based system architecture which allows business process designers to dynamically query and use running instances of real-world services and business applications [38]. More related works and current developments can be found in [39,40].

## 3. Methodology

### 3.1. Requirements Study

**Figure 3.**
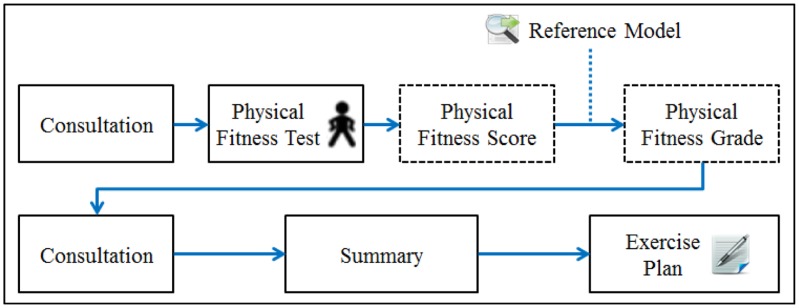
Workflow for generating an exercise plan based on a fitness test.

Domain knowledge encompasses the breadth of knowledge within a field. Developing a knowledge engine to generate pragmatic, personalized exercise plans requires a prior understanding of the process of plan derivation from the perspective of domain professionals. We collaborated with consultants at MJ Health Screening Center, one of the largest health evaluation centers in Taiwan, to capture the general workflow involved in generating a personalized exercise plan based on fitness tests, as illustrated in Figure 3.

In the consultation phase, an instructor elicits background information by asking some personal questions, as illustrated in Table 1. A fitness test is then conducted to assess the subject's physical status.

The test includes nine activities to evaluate the subject's level of fitness. A typical test conducted in the MJ Health Screening Center is depicted in Table 2.

For each activity, test subjects receive a score from one to five based on the Ministry of Education's fitness reference model [41], with higher scores indicating better performance. This scoring scheme may be modified to fit other models. Table 3 shows the male's recommended scoring table for the activity “3 min up and down stairs”.

An instructor then references the outcomes of the consultation, fitness test, and post-test questionnaire (see Table 4) to generate a personalized exercise plan covering the goal, type, time, intensity, and frequency of each suggested exercise.

We modeled the knowledge and plan generation process of the domain expert into the UFIT ontological knowledge engine (UOKE). The UOKE plays a key role in the UFIT architecture, which comprises the exercise knowledge base and inference module. The exercise knowledge base encapsulates ontologically-represented exercise knowledge acquired from domain experts along with user information including personal profiles and health screening data. The SPARQL Inferencing Notation (SPIN) rules [42] and (SPARQL Protocol and RDF Query Language) SPARQL queries [43] enabled inference module manipulates the knowledge contained in the exercise knowledge base to assist plan generation. The UFIT logical and physical architectures are presented in the following section.

### 3.2. Ontology Engineering

There is no correct way to model a domain using ontology and ontology development is necessarily an iterative process. In this sense ontological engineering furnishes various methodologies such as On-To-Knowledge (OTK) [44], METHONTOLOGY [45], United Process for Ontologies (UPON) [46], and Ontology Development 101 [47], to cite but a few for systematically constructing ontologies.

These methodologies are slightly different yet encapsulate many common features. For the purpose of UFIT development we followed the methodology of Ontology Development 101, which consists of the following iterative steps: determination of the domain and scope of the ontology, reuse of existing ontologies, enumeration of important terms, definition of the classes and the class hierarchy, definition of the properties and creation of instances. Through this iterative process, we are enabled to evaluate whether the designed ontology encapsulates the necessary axioms to answer competency questions and to add new knowledge. For example, the Exercise ontology should be able to derive an appropriate exercise plan that details the type, frequency, duration, and intensity of recommended exercises based on the user's physical condition and personal profile.

**Figure 4.**
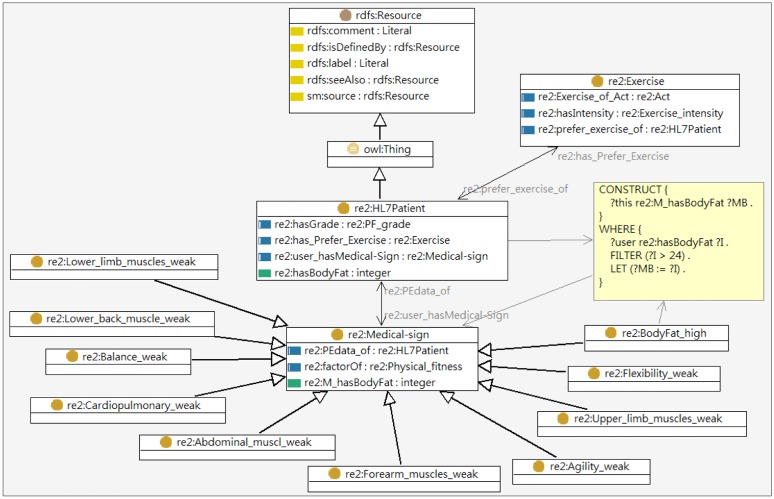
UFIT three ontologies-driven knowledge base.

The UFIT knowledge base is constituted by the exercise, user profile, and health screening ontologies, which defines basic terms and relations comprising the vocabulary of exercise information as well as the constraints for combining terms and relations to define extensions to vocabulary as illustrated in Figure 4. A problem-oriented medical record ontology “HL7-sample-plus-owl” defined by W3C as described in Section 2.3 was reused in constructing these ontologies. The Exercise ontology developed is described below:

Exercise ontology: contains the exercise-related information acquired from domain professionals including:

- Goal of exercise (e.g., cardiopulmonary training, flexibility improvement)
- Type of exercise (e.g., jogging, swimming)
- Time of exercise (e.g., 10∼15 min, 2 rounds, repeat ten times per round)
- Intensity of exercise (e.g., moderate, low, high)
- Frequency of exercise (e.g., 2∼3 times/week, 3∼4 times/week)
- User profile ontology: contains personal information and physical test data including
- Basic information (e.g., name, sex, age, characteristics, preference, interest)
- Personal states (e.g., exercise habit, disabilities, impairments)
- User's preferences (e.g., preferred exercise, preferred time to exercise)
- Health screening ontology: contains comprehensive health-screening information including
- Health-screening data (e.g., physiological data, triglyceride, cholesterol)
- Physical fitness test (e.g., grade 1, grade 2, grade 3, grade 4, and grade 5)

**Figure 5.**
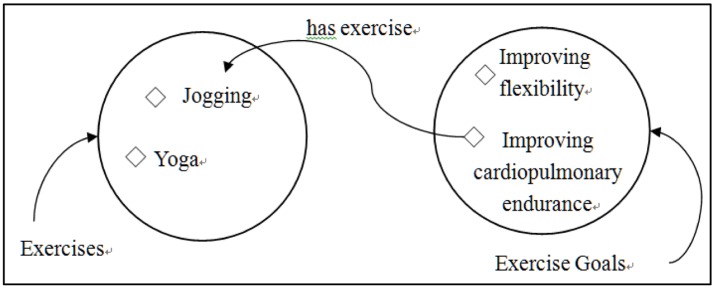
Class-Property-Instance example of UFIT.

Classes, properties, and instances are defined in the ontologies. For example, the instance of “Improving cardiopulmonary endurance” in the class of “Exercise Goals” is associated with the instance of “Jogging” in the class of “Exercise” through the property of “has exercise” as illustrated in Figure 5.

### 3.3. UFIT Logical Architecture

**Figure 6.**
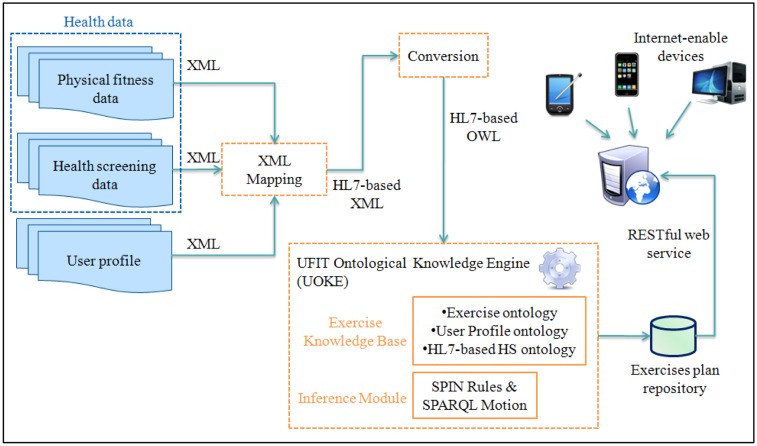
UFIT logical architecture.

Functionally, UFIT is able to infer the appropriate exercise plan which defines the needed exercises through the user's profile, fitness test results, and health screening data. As shown in Figure 6, the process of generating exercise plan is illustrated in the logical architecture. The system is initiated with the data retrieval process:

(1) Data Retrieval: User profile, health screening data, and physical fitness results acquired from health-screening center or medical institution are first retrieved.
(2) Ontology Conversion: TopBraid™ Composer (TBC) [48] tool is used to convert HL7-compliant user profile, physical fitness, and health-screening data into OWL ontologies which will then be used to derive personalized exercise plans through inferencing.
(3) UFIT Ontological Knowledge Engine (UOKE): As the main component of the UFIT architecture, UOKE utilizes the Exercise Knowledge Base that comprises user profile, exercise, and HL7 health-screening ontologies along with constraints and SPIN rules to infer personalized exercise plans using SPARQL query.
(4) Exercise Plan Repository: Storage of personalized exercise plans generated through UOKE.
(5) RSETful Web Service: The generated exercise plans are populated as REST services and can be accessed ubiquitously by Internet-enabled devices.

### 3.4. UFIT Physical Architecture

**Figure 7.**
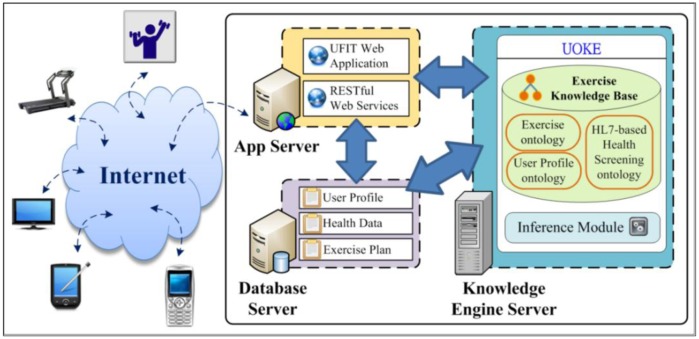
UFIT physical architecture.

The physical representation of UFIT interfaces and system components for the delivery and ubiquitous access of exercise plan generation services is illustrated in Figure 7.

The physical architecture comprises three main components: (1) Knowledge Engine Server; (2) Application Server (App. Server); and (3) Database Server, which are described as follows:

### 3.4.1. Knowledge Engine Server

The UOKE is deployed in a Knowledge Engine Server, which is composed of the Exercise Knowledge Base and Inference Module. The Exercise Knowledge Base in turn is driven by three ontologies: (1) the exercise ontology; (2) the user profile ontology; and (3) the HL7-based health screening ontology.

**Figure 8.**
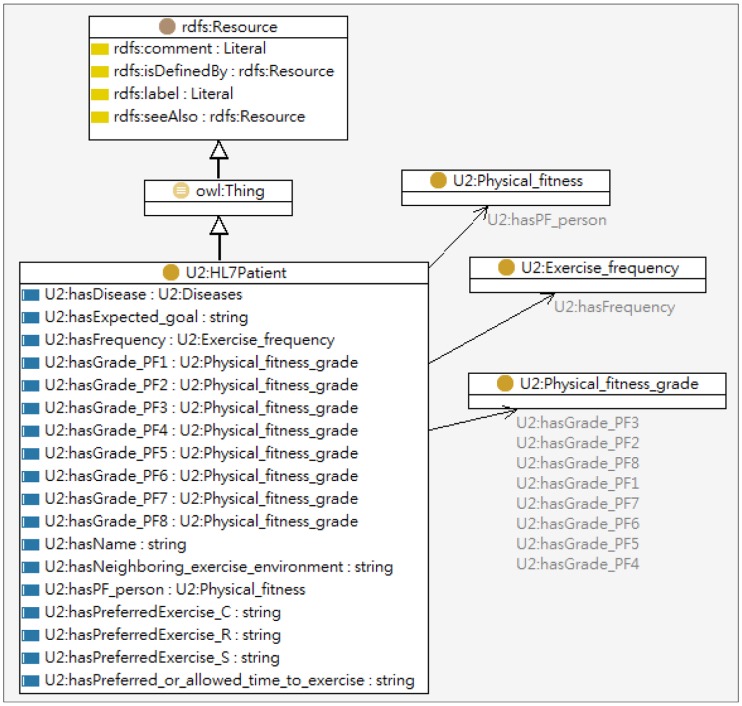
The partial model of user profile ontology.

The knowledge base serves as a knowledge source needed for inferencing personal exercise plans including knowledge acquired from professionals and the user's health status. The User Profile ontology includes the basic information (e.g., name, sex, age, characteristics, preference, interest, *etc.*) and the personal states (e.g., current exercise habit, disabilities, impairments, *etc.*) of an individual. The partial user profile ontology model is shown in Figure 8.

**Figure 9.**
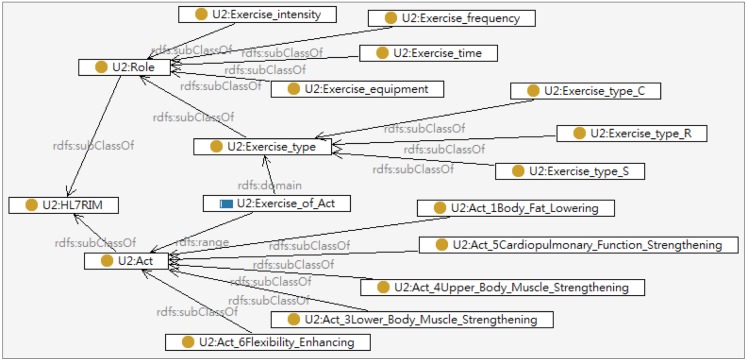
The partial model of Exercise ontology.

The data that describes personal conditions including health screening data, physical fitness tests and the fitness conditions are modeled by the HL7-based Health Screening ontology as illustrated in Figure 6. The health screening data mainly records the features of body composition including body fat, triglyceride, cholesterol, and lipoprotein while the physical fitness tests covers the resulting grades of physical tests shown in Table 2. The Exercise ontology models the characteristics of exercises including the exercise type, intensity, required equipment, improving items *etc*. All exercises are classified into three types in accordance with training goals: Cardiopulmonary training (U2:Exercise_type_C), Resistance training (U2:Exercise_type_R), and Stretch training (U2:Exercise_type_S). The exercises associated with improving items such as lower body muscle strengthening, cardiopulmonary function strengthening, flexibility enhancing, *etc.*, are also modeled in Exercise ontology as shown in Figure 9.

The Inference Module is comprised of two components: (1) SIPN rules and (2) SPARQLMotion [49]. The knowledge acquired from the domain professionals is mostly encoded in SPIN rules. SPARQLMotion provides a platform for drawing inferences on SPIN-encoded knowledge. The process for generating a personalized exercise plan comprises four stages:

1. Determine suitable exercise goals based on user's health status indicated by the health screening data and physical fitness tests.
2. Retrieve available exercises for achieving goals.
3. Filter the inappropriate exercises based on user's profile
4. Formulate the final exercise plan.

For example, suppose that a person has a body fat value of 30. By executing the SPARQL query, the first rule (SPIN rule 1) would suggest that the person's body fat is higher than 24 while the second rule will infer that the person has a medical condition (“high-body-fat”). The third (SPIN rule 3) then creates an exercise goal (“reduce-body-fat”). Once the goal has been determined, a personalized exercise plan can be derived from in-depth inferencing according to the person's conditions. As shown in Table 5, the fourth (SPIN rule 4) finally generates a proposed exercises (“aerobic exercise”) by filtering out water sports because the user is incapable of swimming.

### 3.4.2. Application Server in UFIT

The Application Server is a software framework that provides an environment for running and delivering UFIT applications and services. The server-side UFIT Web Application allows users to maintain data stored in the Database Server while the REST services enable users to access their personal exercise plans ubiquitously.

**Figure 10.**
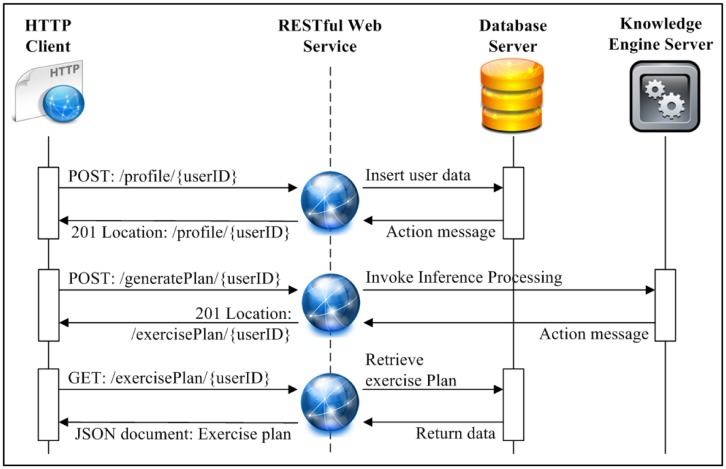
The usage example of REST Service.

- UFIT Web Application provides a Graphical User Interface (GUI) through which users maintain their physical fitness data, health screening data, and user profile.
- REST services in UFIT provide an abstraction for publishing information and serve as an efficient mechanism for communicating with Database Server and Knowledge Engine Server as shown in Figure 10. The REST services can be easily deployed via simple methods of Hypertext Transfer Protocol (HTTP) such as POST, GET, PUT and DELETE and produce discretely formatted responses (typically in XML or JavaScript Object Notation (JSON) with no dangling TCP connection). By leveraging REST services, UFIT is capable of offering ubiquitous access through virtually any Internet-enabled devices.

### 3.4.3. Database Server in UFIT

The Database Server is a repository for the User Profile, Health Data, and Exercise Plan. A user profile includes general information such as age, interests, skills, *etc*. The Health Data stores data from the user's physical fitness test and health screening. The Exercise Plan stores the user's personalized plan including: the goal, type, duration, intensity and frequency of exercise.

## 4. Implementation and Usage Scenarios

### 4.1. Hardware and Software Configurations

UFIT was implemented on Microsoft Windows Server 2003 Standard with TopBraid™ Composer - Maestro Edition (TBC-ME). Apache Tomcat 7.0 and Microsoft SQL Server™ 2008 Express were used for the deployment of application server and database server, respectively.

TBC-ME was adopted for the development of Knowledge Engine (UOKE) because it provides a friendly visual environment for ontology editing. Furthermore, it furnishes a knowledge-based framework for integrating inference engines such as Pellet, Jenna, and TopSPIN. The ability of running multiple inference engines through hybrid inference-chaining shortens the UFIT development cycle. The REST services were coded in java within the Jersey RESTful web services framework [50] incorporated with JavaServer Pages (JSP) technology and Microsoft SQL Server Java DataBase Connectivity (JDBC) Driver. Jersey RESTful web services framework is open source and provides its own Application Programming Interfaces (APIs) to simplify REST services implementation. JSP was used as an efficient means to create dynamic web content while JDBC provides APIs for connecting UFIT Database server. The overall hardware and software configurations of UFIT are summarized in Table 6.

### 4.2. UOKE Knowledge Engine Development

UOKE Knowledge Engine comprises the Exercise Knowledge Base and Inference Module as mentioned in section 3.4.1. The Exercise Knowledge Base encapsulates the models of knowledge acquired from domain experts and users' health status. It is encoded in SPIN and serves as backbone for generating exercise plans through inference.

**Figure 11.**
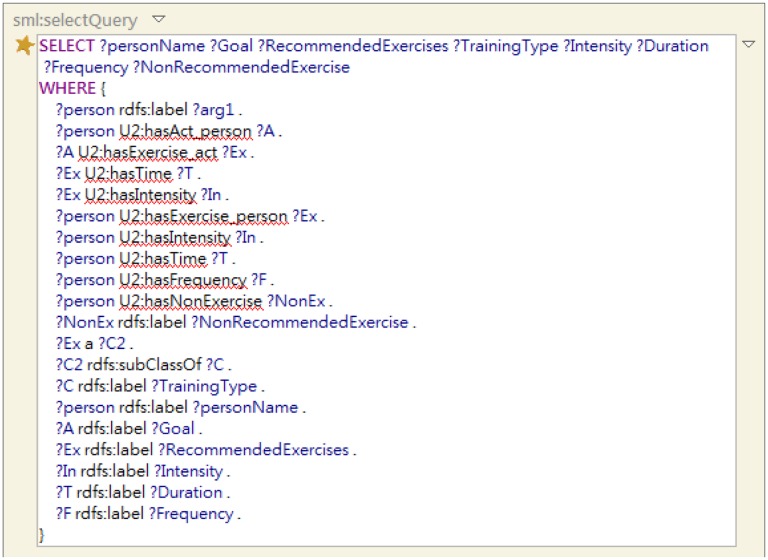
An example of SPARQL query sentence.

**Figure 12.**
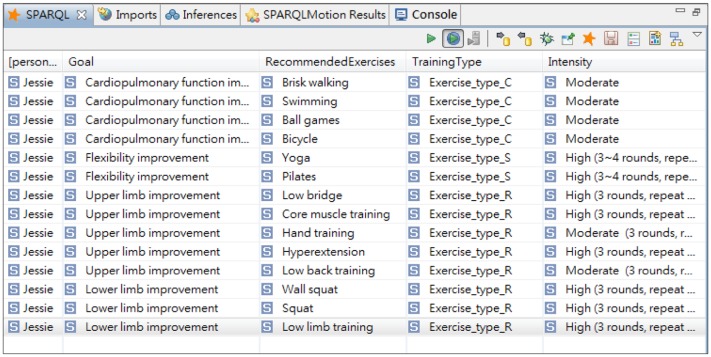
An example of SPARQL query result, Jessie's exercise plan.

The SPARQL query serves as a structural query language to retrieve inference results, the exercise plans, as depicted in Figure 11. An example of generated exercise plan by using SPARQL query is shown in Figure 12. The TBC-ME embedded rule-based reasoner TopSPIN [51] which supports inference with SPIN rules and SPARQL query is applied in UFIT Inference Module.

### 4.3. Usage Scenario and Demonstration

**Figure 13.**
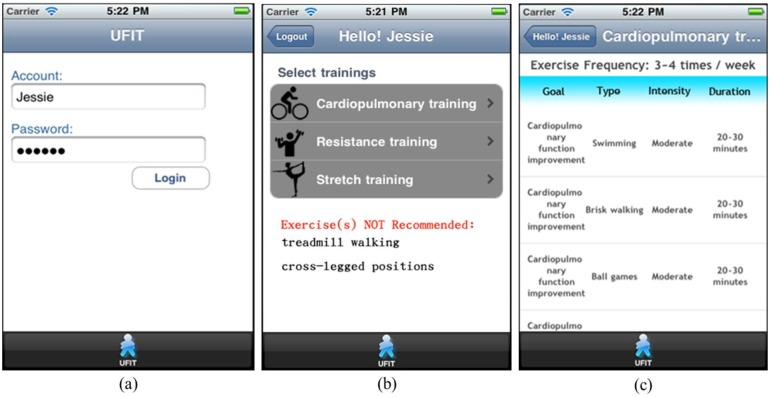
(**a**) UFIT login page; (**b**) Customized exercise plan generated; (**c**) Cardiopulmonary training in the customized exercise plan.

Jessie has an annual health assessment including a fitness test. While traveling on business, she initiates the UFIT web service using her mobile phone, as illustrated in Figure 13a. UFIT then extracts her profile and health data to generate a customized exercise plan, as shown in Figure 13b. According to her health status, the system recommends three areas of exercise: cardiopulmonary, resistance, and stretching, but advises against certain exercise types, such as treadmill walking, because Jessie's medical history indicates she has been suffering from peripheral neuropathy, which can affect balance, placing her at greater risk of falling. Jessie selects the cardiopulmonary training. The recommended exercises associated with the selection are then generated, as depicted in Figure 13c.

As Jessie follows her exercise plan, she can update her fitness through self-assessment. A new plan will then be derived in accordance with her updated fitness status.

### 4.4. Usability Evaluation—Case Study

Usability is a critical factor in determining the ability and willingness of people to use any new technology. In this section, we present a case study conducted at Taipei's MJ Health-screening Center to demonstrate the usability of UFIT and compare system-generated plans against plans prescribed by on-site professionals.

Table 7 presents the fitness evaluation of a volunteer, as prepared by a staff member at the health-screening center. Based on the evaluation and the patient's personal information, an on-site professional then prescribed an exercise program, as illustrated in Table 8.

For benchmarking purposes, the same set of data, including the participant's fitness evaluation and personal profile (e.g., medical history), is used as input for UFIT. Table 9 shows the UFIT-generated exercise plan. In the categories of resistance and stretch training, the exercises recommended by the two plans are essentially identical. As for cardiopulmonary training, the manually-generated plan suggests jogging, spinning and brisk walking while UFIT recommends swimming, bicycling, and brisk walking. Consultation with the on-site professional regarding this discrepancy revealed that, in addition to the patient's physical and health status, she also considered the participant's living environment, which was conducive to jogging and spinning. The health consultant agreed that swimming and bicycling would actually be less risky for a patient with a grade 3 cardiopulmonary evaluation, but the fact that the patient had been playing basketball weekly indicated that he should be able to perform the prescribed exercises without problems.

UFIT strictly complies with professional practices as defined in the knowledge base, and was thus found to generate plans with greater consistency and less flexibility than those generated manually by the health-screening center staff. The manually-generated plans provided more contextually-aware plans which may also expose their clients to injury.

### 4.5. System Usability Evaluation

System usability/readability was evaluated through a questionnaire distributed to 25 general users. Their respective profiles are illustrated in Tables 10.

**Figure 14.**
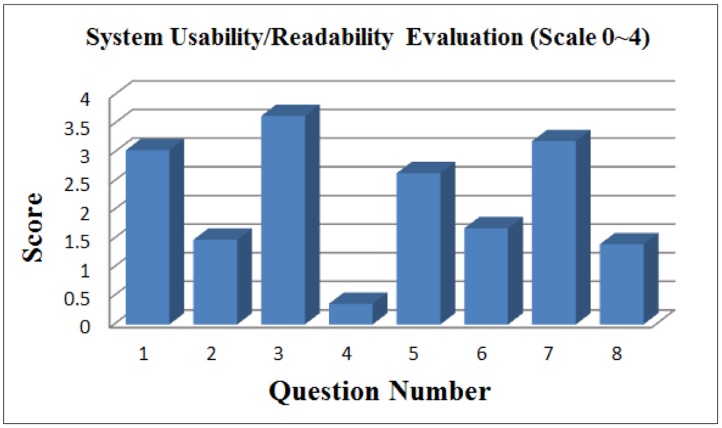
UFIT usability/readability evaluation for users.

Figure 14 shows the results of usability/readability obtained from the questionnaires for general users. In our design of the questionnaire, a question is usually followed by a reversed question (e.g., even number questions) to reveal facts in two opposite sides. From the figure it may be argued that the usability/readability of the UFIT is reasonably high.

The question numbers in Figure 14 stand for:

1. The exercise plan generated by UFIT suits my needs.
2. The exercise plan generated by UFIT is not proportional to my needs.
3. UFIT was easy to use.
4. I would need a technical person to assist me.
5. UFIT has potential in improving my physical conditions.
6. UFIT does not seem to be a promising tool for improving my physical conditions.
7. The features offered by UFIT are sufficient
8. The features offered by UFIT are inadequate

## 5. Conclusions and Future Work

Exercise helps people improve their health, energy and confidence. The ideal exercise program varies from person to person based on individual health conditions and medical history, but many practitioners still take a ‘one size fits all’ approach to recommending exercise regimens, and many practitioners offer inconsistent recommendations based on inconsistent judgment, which can result in inappropriate exercise programs.

This paper describes the design and development of an ontology-based web service (UFIT) to generate personalized exercise program consistently based on personal health data (physical fitness and health screening) and profile (preference). UFIT addresses interoperability issues in health and personal data by adopting the international standard HL7 as the input format. Furthermore, UFIT was developed and deployed using the REST architecture, allowing for ubiquitous access via any Internet-enabled device. Table 11 presents a comparison with similar studies discussed in Section 2 to highlight the advantages and contributions of the proposed UFIT.

While the comparison might not be comprehensive, it provides an overview of the differences between the UFIT and other similar systems to our best knowledge. With the advent of smart devices and the emergence of technologies including Global Positioning System (GPS), ubiquitous sensor networks (USNs), 3G/4G data networks, and radio frequency identification (RFID), smart spaces can be established to deliver context-aware services [52]. The next reasonable step for UFIT development would be to integrate context-aware services. For example, location-aware services could be developed to adapt the service content and presentation to each individual user and his/her current context of use [53]. With location-aware services in place, UFIT could generate customized exercise plans which not only consider the user's profile and health data but also the user's location and current local weather conditions.

## Figures and Tables

**Table 1.** Typical consultation questions.

**No**	**Question**
**1**	Where do you live?
**2**	Where do you work?
**3**	Do you exercise regularly (2∼3 times/week)?
**4**	To continue with question number 3, what type of exercise?
	What is the duration of your exercise?
**5**	Have you ever had surgery?
**6**	Have you ever suffered from arthrosis injury?
**7**	Do you have the cardiovascular disease?
**8**	Do you take any drug?
**9**	Do you smoke habitually?

**Table 2.** Fitness test items.

**No**	**Activity**	**Fitness Assessment**
**1**	3 min up and down stairs	Cardiovascular endurance
**2**	Sit and reach	Flexibility
**3**	1 min curl ups	Muscular strength/endurance
**4**	Vertical jump 2 times	Muscular strength/endurance (explosive force of lower limb)
**5**	Dominant grip strength test (squeezes grip strength dynamometer with maximum isometric effort for about five seconds)	Grip muscular strength/endurance
**6**	Back and leg strength test with back dynamometer	Back and leg muscular strength
**7**	Stork Balance Stand Test	Balance
**8**	20 s shuttle run	Agility
**9**	Body Mass Index (BMI)	Body composition

**Table 3.** Recommended scoring matrix for male's “3 min up and down stairs”.

**Age**	**Grades**				

	**1**	**2**	**3**	**4**	**5**
6	∼47.7	47.8 ∼ 54.8	54.9 ∼ 58.6	58.7 ∼ 65.0	65.1 ∼
7	∼50.2	50.3 ∼ 56.5	56.6 ∼ 59.9	60.0 ∼ 68.5	68.6 ∼
8	∼51.1	51.2 ∼ 55.7	55.8 ∼ 59.7	59.8 ∼ 65.9	66.0 ∼
9	∼52.7	52.8 ∼ 57.8	57.9 ∼ 63.4	63.5 ∼ 68.7	68.8 ∼
10	∼52.2	52.3 ∼ 55.3	55.4 ∼ 59.6	59.7 ∼ 65.9	66.0 ∼
11	∼52.4	52.6 ∼ 56.9	57.0 ∼ 61.5	61.6 ∼ 66.4	66.5 ∼
12	∼51.1	51.2 ∼ 58.0	58.1 ∼ 62.7	62.8 ∼ 70.5	70.6 ∼
13	∼54.2	54.3 ∼ 59.0	59.1 ∼ 64.1	64.2 ∼ 69.7	69.8 ∼
14	∼55.0	55.1 ∼ 59.6	59.7 ∼ 63.8	63.9 ∼ 68.4	68.5 ∼
15	∼52.4	52.5 ∼ 57.8	57.9 ∼ 60.3	60.4 ∼ 69.5	69.6 ∼
16	∼51.9	52.0 ∼ 56.4	56.5 ∼ 60.3	60.4 ∼ 65.0	65.1 ∼
17	∼51.9	52.0 ∼ 56.9	57.0 ∼ 60.3	60.4 ∼ 68.0	68.1 ∼
18	∼50.3	50.4 ∼ 54.3	54.4 ∼ 59.0	59.1 ∼ 66.4	66.5 ∼
19	∼50.6	50.7 ∼ 54.8	54.9 ∼ 59.7	59.8 ∼ 64.6	64.7 ∼
20-25	∼50.6	50.6 ∼ 55.8	55.9 ∼ 59.9	60.0 ∼ 67.3	67.4 ∼
26-30	∼49.1	49.2 ∼ 53.0	53.1 ∼ 56.4	56.5 ∼ 62.1	62.2 ∼
31-35	∼48.7	48.8 ∼ 53.2	53.3 ∼ 57.0	57.1 ∼ 63.5	63.6 ∼
36-40	∼49.0	49.1 ∼ 53.8	53.9 ∼ 58.9	59.0 ∼ 65.9	66.0 ∼
41-45	∼49.9	50.0 ∼ 54.1	54.2 ∼ 59.7	59.8 ∼ 66.5	66.6 ∼
46-50	∼50.5	50.6 ∼ 55.0	55.0 ∼ 59.3	59.4 ∼ 66.1	66.2 ∼
51-55	∼50.6	50.7 ∼ 55.7	55.8 ∼ 60.7	60.8 ∼ 67.6	67.7 ∼
56-60	∼49.5	49.6 ∼ 55.7	55.8 ∼ 60.9	61.0 ∼ 69.5	69.6 ∼
61-65	∼47.6	47.7 ∼ 53.5	53.6 ∼ 60.1	60.2 ∼ 66.1	66.2 ∼

**Table 4.** Post-test questionnaire.

**No**	**Question**
**1**	Expected goal (Weight-loss, flexibility improvement, *etc.*)
**2**	Available exercise environment (Gym, sports field, *etc.*)
**3**	Preferred/allowed time to exercise (Daily after 5pm, on weekends, *etc.*)
**4**	Preferred exercises (Jogging, swimming, basketball, *etc.*)

**Table 5.** A simple illustration for the inference module.

**SPARQL query**	SELECT ?user ?Goal
	WHERE { ?User hasBodyFat ?I. }
**SPIN rule 1**	CONSTRUCT {?user hasHighBodyFat ?I.}
	WHERE{ ?this hasBodyFat ?I.
	FILTER (?I > 24). }
**SPIN rule 2**	CONSTRUCT { ?I value_of_MedicalSign ?M. }
	WHERE { ?user hasHighBodyFat ?I. }
**SPIN rule 3**	CONSTRUCT { ?user hasGoal ?G. }
	WHERE { ?G hasMedicalSign ?M.
	?I value_of_MedicalSign ?M.
	?user hasHighBodyFat ?I. }
**SPIN rule 4**	CONSTRUCT { ?user hasPreferredExercise ?E. }
	WHERE { ?user hasGoal ?G.
	?G rdfs:label “reduce-body-fat”.
	?E hasTypeAerobics “Aerobic”.
	FILTER (?user hasAbilitySwimming “false”.
	?E hasTypeWatersports “false”). }

**Table 6.** UFIT Hardware and software configuration.

**Hardware environment configuration**	
CPU	Intel(R) Xeon(R) E5310 1.6GHz
Memory	4.0 GB
Operation System	Microsoft Windows Server 2003 Standard Edition

Runtime and implementation configuration	

Database	Microsoft SQL Server 2008 Express Edition
Application Server	Apache Tomcat 7.0
Java Platform	Java SE Development Kit 1.6
REST Framework	Jersey 1.18
JDBC Driver	Java DataBase Connectivity 3.0
Knowledge Engine Framework	TopBraid™ Composer - Maestro Edition

**Table 7.** Physical fitness evaluation.

**Test Items**		**Results**		

		**Scores**	**Grades (1∼5)**	**Unit**
**1**	3 min up and down stairs	54.2	3	Index
**2**	Sit and reach	44	5	cm
**3**	1 min curl ups	28	4	times/min
**4**	Vertical jump 2 times	45	4	cm
**5**	Dominant grip strength test	23.5	1	kg
**6**	Back and leg strength test with back dynamometer	56.5	1	kg
**7**	Stork Balance Stand Test	10	1	second
**8**	20 s shuttle run	35	4	times/20 s

**Table 8.** Manually-generated personalized exercise plan (Plan A).

**Summary**			
Date: 2013/01/23 Time: 2:30 p.m. Frequency: 3∼4 times/ week Goal: Exercise regularly			
**Cardiopulmonary training**			
No	Type of exercise	Intensity of exercise	Time/duration of exercise
1	Jogging	20 minutes up	Moderate
2	Spinning	20 minutes up	Moderate
3	Brisk walking	20 minutes up	Moderate
**Resistance training**			
No	Type of exercise	Intensity of exercise	Time/duration of exercise
1	Core muscle training	—	—
**Stretch training**			
No	Type of exercise	Intensity of exercise	Time/duration of exercise
1	Pilatisi	—	—
2	Larger muscle groups	—	—

**Table 9.** Personalized exercise plan generated by UFIT (Plan B).

**Exercise plan**			
Date: 2013/08/17 Time: 2:30 pm Frequency: 3∼4 times/ week Goal: Exercise regularly			
**Cardiopulmonary training**			
No	Goal of exercise	Type of exercise	Intensity of exercise
1	Cardiopulmonary function improvement	Swimming	Moderate
2	Cardiopulmonary function improvement	Brisk walking	Moderate
2	Cardiopulmonary function improvement	Bicycle	Moderate
**Resistance training**			
No	Goal of exercise	Type of exercise	Intensity of exercise
1	Upper limb improvement	Core muscle training	High (15∼20 times)
2	Upper limb improvement	Low bridge	High (15∼20 times)
3	Upper limb improvement	Hand training	Moderate (12∼15 times)
4	Upper limb improvement	Hyperextension	High (15∼20 times)
5	Upper limb improvement	Low back training	Moderate (12∼15 times)
6	Lower limb improvement	Wall squat	High (15∼20 times)
7	Lower limb improvement	Squat	High (15∼20 times)
8	Lower limb improvement	Low limb training	High (15∼20 times)
**Stretch training**			
No	Goal of exercise	Type of exercise	Intensity of exercise
1	Flexibility improvement	Pilatisi	High (3∼4 rounds, repeat fifteen minutes per round)
2	Flexibility improvement	Yoga	High (3∼4 rounds, repeat fifteen minutes per round)

**Table 10.** User profiles.

**Personal information**	**Number**	**%**
*Age*		
20–30	14	56.0
30–40	8	32.0
40 or above	3	12.0
Total	25	100.0
*Gender*		
Male	14	56.0
Female	11	44.0
Total	25	100.0
*Experience of using Smart Mobile Device*		
Yes	21	84.0
No	4	16.0
Total	25	100.0
*Exercise habit (Average times per week)*		
1 or below	12	48.0
2	11	44.0
3 or above	2	8.0
Total	25	100.0

**Table 11.** Comparison of UFIT and other proposed systems.

	Service domain	Service objective	Personalization Service	Ubiquitous Service	Web services based architecture
Kanellopoulos [15]	e-Commerce	Produces recommendation	Yes	No	No
Liao *et al.* [16]	e-Library	Library services recommendation	Yes	No	No
Vesin *et al.* [17]	e-Learning	Tutoring system	Yes	No	No
Riaño *et al.* [19]	Healthcare	Clinical decisions	Yes	No	No
Isern *et al.* [21]	Healthcare	Clinical guidelines	No	No	No
Valls *et al.* [22]	Home care	Organizational knowledge structuring	No	No	No
García-Crespo *et al.* [23]	Healthcare	Clinical decisions	No	No	No
Alexandrou *et al.* [24]	Healthcare	Treatment plans	Yes	No	No
**UFIT**	**Healthcare**	**Exercise plans**	**Yes**	**Yes**	**Yes**
